# COVID-19 vaccine acceptability and determinants among pregnant mothers attending antenatal care services at Debre Markos town public health institutions, Debre Markos Northwest Ethiopia: mixed study

**DOI:** 10.11604/pamj.2022.41.293.32618

**Published:** 2022-04-12

**Authors:** Bewket Yeserah Aynalem, Misganaw Fikrie Melesse, Liknaw Bewket Zeleke

**Affiliations:** 1 Department of Midwifery, Debre Markos University, Debre Markos, Ethiopia,; 2 School of Women´s and Children´s Health, University of New South Wales, Sydney, Australia

**Keywords:** COVID-19, COVID-19 vaccine, Debre Markos, pregnant mothers, Ethiopia

## Abstract

**Introduction:**

coronavirus is a communicable disease that produces severe morbidity and mortality in the globe and more than three million people died due to COVID-19. Pregnant mothers are at higher risk of COVID-19 viral infection, with great morbidity and mortality. Thus, the purpose of this research is to assess the level of COVID-19 vaccine acceptability, determinants, and hesitancy among pregnant mothers attending antenatal care at Debre Markos town, public health institutions, Debre Markos, Northwest Ethiopia.

**Methods:**

a mixed study was conducted among 350 pregnant mothers attending antenatal care at Debre Markos town health institutions and the participants were selected by consecutive sampling techniques. The collected data were entered into EPI Info version 7 and then exported to SPSS version 25 for data cleaning and analysis. The level of COVID-19 vaccine acceptability was determined through descriptive statistics, whereas its determinants were identified by binary logistic regression analyses. Variables with p-value < 0.05 in multivariable were considered as significantly associated factors. The qualitative data were collected by an unstructured interviewer guide using in-depth interview data collection methods. Study participants were selected purposively until the required data was saturated. The data was analysed under selected themes based on the guide and summarized manually.

**Results:**

sixty-five (18.5%) of the respondents accept the COVID-19 vaccine [95% CI: 13, 23]. Maternal age [AOR: 3.281 (95% CI: 1.184, 9.092)], chronic medical illness [AOR: 0.170 (95% CI: 0.051, .562)], information about COVID-19 vaccine [AOR: 4.063 (95% CI: 1.462, 11.293)], pregnancy-induced medical conditions [AOR: 4.131 (95% CI: 1.055, 16.183) were identified as significant determinants of COVID-19 vaccine acceptability. From the quantitative wing. The qualitative finding implied that misconception, fear of medical complications, lack of trust in its effectiveness, and religious constraints were the common reasons for vaccine hesitancy.

**Conclusion:**

COVID-19 vaccine acceptability by pregnant mothers attending ANC at Debre Markos town public health institutions is very low. The health care providers and health extension workers shall create information about the COVID-19 vaccine on its importance and side effects.

## Introduction

Coronavirus (COVID-19) is a communicable disease that produces severe morbidity and mortality around the globe and more than three million people died due to COVID-19 [1]. COVID-19 infection and risk of death is increasing in people who have had chronic diseases like diabetes mellitus, hypertension, and other immunity suppressive diseases [2, 3]. Globally, different measurement is undertaken to tackle the increased spread of the COVID-19 and activities were awareness creation about the behaviour of the disease, delivering mask and handwashing materials as well as antiseptic solution and social mobilization for vaccine nation [4]. Pregnant mothers are at higher risk of COVID-19 viral infection, with great morbidity and mortality [5]. Due to this complication, different organizations like the World Health Organization recommended the COVID-19 vaccine for pregnant mothers [6, 7]. The vaccine acceptance level is varying from country to country, even different in the same country in different regions [8-10]. Currently, COVID-19 vaccine acceptability is in question due to rumours gained from the community and different mass Media related to the vaccine content, side effects, and aims [11, 12]. Therefore, creating awareness related to the natural properties and importance of the COVID-19 vaccine is essential to increase the coverage of vaccine utilization [13].

The coverage of COVID-19 vaccine utilization is expected to be from 70 to 80 percent in every country´s population and this decreases the new infection of the disease. Currently, the COVID-19 vaccine acceptability in pregnant mothers is also varied from country to country [14, 15]. Ethiopia uses the World Health Organization´s recommendation for COVID-19 vaccine utilization and the Ethiopian ministry of health has identified maternal services to improve maternal and child health and decrease COVID-19 infections [16]. Despite the Ethiopian ministry of health allowing and using the World Health Organization´s recommendation, the level of COVID vaccine acceptability, determinants, and reasons for COVID vaccine hesitancy is not well known. There is one study done in Ethiopia which showed vaccine acceptability as 70.9% [17]. But this single study is not determining the exact figure of the acceptability and it didn´t explore the reasons that make the pregnant mothers hesitate to utilize the COVID-19 vaccine. Thus, the purpose of this research is to assess the level of COVID-19 vaccine acceptability, determinants, and hesitancy among pregnant mothers attending antenatal care (ANC) at Debre Markos town, public health institutions, Debre Markos, Northwest Ethiopia.

## Methods

**Study area and period:** the study area was Debre Markos town, Amhara region, Northwest Ethiopia from September 1^st^, 2021, to October 30^th^, 2021. Debre Markos town is the capital city of East Gojjam Zone in Amhara regional State, Northwest Ethiopia, which is located at 300 km from Addis Ababa, the capital city of Ethiopia, and 265 km from Bihar Dar, the capital city of Amhara region. Debre Markos town has six governmental health institutions and five non-governmental clinics that give different reproductive health services in the town.

**Study design:** a facility-based mixed study design was used.

**Study participants:** the source population was all pregnant mothers who were attending ANC services in Debre Markos town public health institution with age above 18 years. The study population was all pregnant mothers who were attending ANC services during the data collection period in Debre Markos town public health institution with age above 18 years. Those who came from the study area for a temporary checkup and who were critically ill during the data collection period were excluded from the study.

### Sample size

***Quantitative:*** the sample size was calculated based on the assumption of the single population proportion formula. The magnitude of COVID-19 vaccine acceptance was 70.9% [17] and a 5% margin of error was used.


$$\begin{array}{l} \text{initial sample size for knowlede}={\left(Z\frac{a}{2}\right)}^{2}*\frac{P\left(1-p\right)}{w^{2}}\\ =\left[{\left(1.96\right)}^{2}*0.709\left(1-0.709\right)/{\left(0.005\right)}^{2})\right]=318 \end{array}$$

We have added a non-response rate of 10% and 318*0.10=32. The final sample size was 350.

***Qualitative:*** the qualitative study was conducted among purposively selected participants in the health institutions. The overall sample size for the qualitative data was mainly determined by the information saturation level.

### Sampling techniques

***Quantitative:*** simple random sampling was employed to select 3 public health institutions from all public health institutions at Debre Markos town and the sample size was proportional allocated to the selected health institution. The final study participant was added to the sample consecutively.

***Qualitative:*** the participants required for the qualitative interview were selected by convenience non-random sampling technique. For an in-depth interview, mothers were selected from the health study area. Required number of study participants was selected by convenience non-random sampling method to participate in the in-depth interview during and after quantitative data collection.

### Study variables

***Dependent variable:*** coronavirus vaccine acceptability.

***Independent variables:*** socio-demographic characteristics; health condition and obstetric characteristics and awareness about COVID-19.

### Data collection tool and data quality control

***Quantitative:*** an interviewer-administered structured and pre-tested questionnaire was used by three trained BSc nurses and one MSc health nurse supervisor. One-day data collection training was given for both data collectors and supervisors. The questionnaire was first prepared in English and translated to local language and then again translated back to English. A pretest was conducted on 5% (10) of the sample size in Lumamie hospital and the necessary correction was taken accordingly.

***Qualitative:*** the qualitative data was collected by an unstructured interviewer guide using in-depth interview data collection methods. Data was collected through in-depth interviews to address our objective. Study participants were selected purposively until the required data was saturated.

### Data processing and analysis

***Quantitative:*** the collected data were entered into EPI Info version 7 and then exported to SPSS version 25 for data cleaning and analysis. Descriptive statistics were computed to describe the study population about relevant variables. Binary logistic regression was used to identify factors associated with the outcome variable. Independent variables that showed P-value < 0.25 in the bivariate logistic regression analysis were included in the multivariable logistic regression analysis. Finally, variables with P-value < 0.05 at a 95% confidence interval were declared as significantly associated with the outcome variable. The strength & direction of the association was interpreted based on the adjusted odds ratio.

***Qualitative:*** in addition to recording in audio file field notes was be taken for each interview. The audio file, consent form, field note, and in-depth enrolment form were coded properly and consistently. Every day, every recorded audio file was checked for its appropriateness in terms of proper coding, signed consent, clear audibility, level of depth questioning, and consistency of the codes was given for the audio file, consent, and field note. Finally, data were analyzed under selected themes based on the guide and summarized manually.

**Ethical acceptability:** the ethical acceptability and permission were obtained from the health department of Debre Markos town. Written informed consent was obtained from each study participant after explaining the purpose and ethical process of the study. Finally, the women were interviewed in private rooms independently, the data were kept anonymous and the participant had the right to withdraw from the study without restriction.

## Results

### Quantitative

**Socio-demographic characteristics:** all sampled study participants gave a response to the questionnaire, giving a response rate of 100%. About 245 (70%) of the study participants were married and the majority of the respondents (97.5%) were Orthodox Christians (Table 1).

**Table 1:** socio-demographic characteristics among pregnant mothers (n=350) attending ANC at Debre Markos town public health institutions, Northwest Ethiopia, 2021

Variable	Frequency	Percent
**Maternal age**		
18-30	193	55
31-45	157	45
**Marital status**		
Married	245	70
Others*	105	30
**Religion**		
Orthodox	341	97.5
Muslim and protestant	9	2.5
**Educational status**		
No formal education	93	26.6
Primary education	72	20.6
Secondary education	99	28.1
College and above	86	24.6
**Occupation**		
Housewife	49	14
Self-employee	149	42.5
Private employee	107	30.5
Government employee	45	13

* Single, divorced, and widowed

**Health condition and obstetric characteristics:** the majority of the respondents (85.4%) were free from any chronic medical problems and only 4% were tested for COVID-19. About 107 (30.6%) of the study participants were grand multipara (Table 2).

**Table 2:** medical and obstetric characteristics, awareness about COVID-19 and acceptability of COVID-19 vaccine among pregnant mothers (n=350) attending ANC services at Debre Markos town public health institutions, Northwest Ethiopia, 2021

Variable	Frequency	Percent
**Chronic medical condition**		
no	299	85.4
yes	51	14.6
**Tested for COVID-19**		
No	336	96
Yes	14	4
**Covid test result**		
Positive	3	21
Negative	11	79
**First ANC visit**		
No	206	59
Yes	144	41
**Pregnancy-induced medical problem**		
No	219	62.6
Yes	131	37.4
**Parity**		
0-5	243	69.4
>5	107	30.6
**Had information about COVID vaccine**		
No	126	36
Yes	224	64
**Source of information**		
Health care providers	28	12.5
Media	131	58.5
Others*	65	29
**Accepted for COVID-19 vaccination**		
No	285	81.4
Yes	65	18.6

* friends and family

**Awareness about coronavirus and COVID-19 vaccine acceptability:** about 224 (64%) of respondents had information about COVID-19 and majorities (58.5%) got the information from the media. Sixty-five (18.6%) of the respondents accept the COVID-19 vaccine [95% CI: 13, 23] (Table 2).

**Determinants of COVID-19 vaccine acceptability:** in binary logistic regression, maternal age, chronic medical illness, tested for COVID-19, Parity, Income, information about the COVID-19 test, Pregnancy-induced medical conditions, and the number of ANC visits were the determinants of COVID-19 vaccine acceptability (P-values<0.25). Again, in multivariable logistic regression, maternal age [AOR: 3.281 (95% CI: 1.184, 9.092)], chronic medical illness [AOR: 0.170 (95% CI: 0.051, .562)], having information about COVID-19 vaccine [AOR: 4.063 (95% CI: 1.462, 11.293)], Pregnancy-induced medical conditions [AOR: 4.131 (95% CI: 1.055, 16.183) were the determinants of COVID-19 vaccine acceptability (P-value ≤0.05) (Table 3).

**Table 3:** bivariable and multivariable analysis of determinants of COVID-19 vaccine acceptability among pregnant mothers (n=350) attending ANC at Debre Markos town public health hospitals, Northwest Ethiopia, 2021

Variable	Accept COVID-19 vaccine		Crude OR[95%CI]	AOR[95%CI]
	**No**	**Yes**		
**Age of mothers**				
18-30	170	23	**1**	**1**
31-45	115	42	**2.699(.1.290, 5.709)**	**3.281(1.184,9.092)**
**Marital status**				
Others*	87	18	0.837(.377,1.860)	.
Married	198	47	1	
**Religion**				
Orthodox	278	63	1	
Muslim and protestant	7	2	1.104 (0.120,10.176)	
**Educational status**				
No formal education	81	12	1	
Primary education	53	19	2.410(.840, 6.909)	
Secondary education	81	18	1.429(.501, 4.077)	
College and above	72	14	1.282(.428, 3.845)	
**Occupation**				
Housewife	45	4	1	
Self-employee	124	25	2.563(.545, 12.056)	
Private employee	79	28	.4.622(.984, 21.717)	
Government employee	36	9	3.095(.545, 17.595	
**Chronic medical condition**				
no	252	47	**1**	**1**
yes	33	18	**2.892507(1.177, 6.699)**	**5.882(1.961,10.417)**
**Tested for COVID-19**				
No	277	59	**1**	1
Yes	9	5	**2.608 (1.54,5.011)**	4.305 (.684, 27.075)
**First ANC visit**				
No	180	26	**1**	1
Yes	105	9	**0. 593(.082, 0.1915)**	.405 **(**.099, 1.654**)**
**Pregnancy-induced medical problem**				
No	194	25	**1**	**1**
Yes	91	40	**3.411(1.671,7.362)**	**4.131(1.055, 16.183)**
**Had information about COVID vaccine**				
No	44	82	**1**	**1**
Yes	21	203	**5.187(2.387,11.074)**	**4.063(1.462, 11.283**
**Source of information**				
Health care providers	24	4	1	
Media	109	22	1.600(.172, 14.904)	
Others**	53	12	1.882(.180, 19.677)	
**Parity**				
0-5	212	31	**1**	1
>5	74	33	**3.049(1.460, 1.460)**	1.990(.784, 5.052)

* single, divorced, and widowed;**friends and family

### Qualitative

**Socio-demographic characteristics:** seventeen pregnant mothers participated from the study area in the in-depth interview on reasons for COVID-19 vaccine hesitancy. Eleven (64.7%) participants were in the age of below 30 and above 18 years and all of the participants were Amhara in ethnicity. The majority of the participants (82%) were Orthodox Christians and the others were Muslims in religion. Around 15 (88.2%) of the mothers were married and the other 2 (11.8%) of the participants were divorced. Around 4 (23.5%) of the participants were unable to read and write and the others can write and read. Around 5 (29.4) of the respondents were housewives, 8 (47%) were government employees and 4 (23.5%) were private workers. About 13 (76.5%) were multi gravida and the others primi gravida.

**Reasons for COVID-19 vaccine hesitancy:** some reasons for COVID-19 vaccine hesitancy were explored. Some participants said, *"I have heard some rumors on the content of the vaccine; I heard that the vaccine contains bad spirit which will affect my daily life”*.(Participant # 4, 7 and 11)

The other two participants had some concerns about the teratogenicity effect of the vaccine. They said that *"I have a fear my baby may have a congenital malformation like deafness, vision problems, and other congenital complications"*.(Participant # 1, 2, 13, 14 and 16)

A 29 years participant had concerns related to the effectiveness of the vaccine and health care providers. She said that *“health care providers that I know are not vaccinated because they may have the information on the effectiveness of the vaccine; if it is effective to prevent the disease, the health care providers may take the vaccine”*.(Participant # 7)

Some participants had concerns about the previous history of drug allergy. They said that *“I have a history of oral and injection drug histories which were manifested by shock. So, if this allergy occurs, my fetus may get trauma due to falling*.(Participant # 2, 3 and 9)

Some also had some issues with the adverse effect of the vaccine during delivery. They said that *“I heard that the vaccine may produce post-partum hemorrhage during delivery”*.(Participant # 5,6,11 and 12)

Some also have concerns about the unknown effect of the vaccine. They said that *“the vaccine may have an unknown effect because the time is too short to know the long term effect of the vaccine and I think it is not well studied"*.(Participant # 7, 15, and 17)

Some also have fear of injection don't believe the drug itself. They said, *"I fear the injection and don't believe the drug. I believe the drug had a substance that makes me addicted for something like smoking and other addiction"*.(Participant # 1, 3, 6 and 7)

Some said that: *“the drug makes me uncontrolled myself and someone may control my brain and use it. So someone may get more power by controlling the peoples’ mind”*.(Participant # 8, 9, 17)

Some had a related it with their religion. They said that *“I believe in God, COVID-19 is the disease for who doesn’t believe in God. So this disease has no power in me.”* (Participant # 7, 13, and 15). Some don't consider it as a disease that is different from the common cold. They said that *"we Ethiopians have an experience COVID related disease like common cold and influenza that made us develop immunity"*.(Participant # 5, 6, and 11)

Some believe feeding behavior protects against COVID-19. *"I believe that Ethiopian people’s food is spicy and had a pepper in it during preparation. So these spicy foods protect Ethiopian people from infection and will kill the COVID-19 if infection occurs."*(Participant # 3, 13, and 16)

## Discussion

A very small number of study participants accepted the COVID-19 vaccine (18.5 [95% CI: 13, 23]). The main reason for this low COVID-19 vaccine acceptance of the mothers might be rumors on COVID-19 vaccine; they fear the vaccine adverse effect and mothers have no clear information about the side effects and the complication of the vaccine. This is supported by the participants who participated in the qualitative study. Again this finding is lower the studies done Ethiopia 48% and (62.6%) [18-20], Kuwait (53.1%) [21], Saudi Arabia (48%) (90.4%) [22, 23], middle eastern population (63.2) [24], Korea (76.5%), [25], Bangladesh (67%) [26], Iraq (61.7%) [27]. The difference may be due to the study population and the difference in socio-economic status as shown from the study done in Korea, Saudi Arabia, and Iraq.

Respondents with increased age were 3.281 times more likely to accept the COVID-19 vaccine. This result is supported by the studies done in Ethiopia [18], Saudi Arabia [28], Bangladesh [26]. This may be due to the fear of death related to COVID-19. As age increases the probability to be infected with COVID-19 will also increase so that the aged people prefer to be vaccinated for COVID-19. Mothers who have chronic medical problems were 5.882 times more likely to accept the COVID-19 vaccine as compared with healthy participants. This is supported by the studies done in Ethiopia [18, 19], Bangladesh [26]. The main justification maybe those who are in chronic medical conditions may get more fear about COVID-19 infection because they may have information about the behavior of COVID-19 which has more impact on patients with a chronic health problem.

Those pregnant mothers with pregnancy-induced medical disease wore 4.131 times more likely to accept the COVID-19 vaccine as compared with pregnant mothers without pregnancy-induced health conditions. No study supports or contradicted this finding but the main reason for this might be pregnant mothers with the pregnancy-induced disease may give more concern to COVID-19 infection since it affects more people with an obstetric and medical health condition. The study participants who had awareness about the COVID-19 vaccine, 4.063 times more likely to accept COVID-19 vaccination as compared with those who had no information on COVID-19 vaccination. This finding is supported by the study done in Ethiopia [18, 20], Saudi Arabia [22] Korea [25]. The main explanation for this might be mothers who had more information about side effects and the benefits of the vaccine may help them to accept the vaccination.

## Conclusion

COVID-19 vaccine acceptability by pregnant mothers attending ANC at Debre Markos town public health institutions is very low. Maternal age, chronic medical illness, having information about the COVID-19 vaccine, pregnancy-induced medical conditions were the determinants of COVID-19 vaccine acceptability. The health care providers and health extension workers shall create information about the COVID-19 vaccine on its importance and side effects.

### What is known about this topic

- *Globally, different measurement is undertaken to tackle the increased spread of the COVID-19;*
- *Pregnant mothers are at higher risk of COVID-19 viral infection, with great morbidity and mortality;*
- *COVID-19 vaccine acceptability is in question due to rumours gained from different Media*.

### What this study adds

- *Maternal willingness towards COVID-19 vaccines is very low and educating the importance of COVID-19 vaccine may increase the acceptability of the vaccine*.
